# Sampling Realistic Protein Conformations Using Local Structural Bias

**DOI:** 10.1371/journal.pcbi.0020131

**Published:** 2006-09-22

**Authors:** Thomas Hamelryck, John T Kent, Anders Krogh

**Affiliations:** 1 Bioinformatics Center, Institute of Molecular Biology and Physiology, University of Copenhagen, Copenhagen, Denmark; 2 The Department of Statistics, The University of Leeds, Leeds, West Yorkshire, United Kingdom; Peking University, China

## Abstract

The prediction of protein structure from sequence remains a major unsolved problem in biology. The most successful protein structure prediction methods make use of a divide-and-conquer strategy to attack the problem: a conformational sampling method generates plausible candidate structures, which are subsequently accepted or rejected using an energy function. Conceptually, this often corresponds to separating local structural bias from the long-range interactions that stabilize the compact, native state. However, sampling protein conformations that are compatible with the local structural bias encoded in a given protein sequence is a long-standing open problem, especially in continuous space. We describe an elegant and mathematically rigorous method to do this, and show that it readily generates native-like protein conformations simply by enforcing compactness. Our results have far-reaching implications for protein structure prediction, determination, simulation, and design.

## Synopsis

Protein structure prediction is one of the main unsolved problems in computational biology today. A common way to tackle the problem is to generate plausible protein conformations using a fairly inaccurate but fast method, and to evaluate the conformations using an accurate but slow method. The main bottleneck lies in the first step, that is, efficiently exploring protein conformational space. Currently, the best way to do this is to construct plausible structures by stringing together fragments from experimentally determined protein structures, a method called fragment assembly. Hamelryck, Kent, and Krogh present a new method that can efficiently generate protein conformations that are compatible with a given protein sequence. Unlike for existing methods, the generated conformations cover a continuous range and come with an associated probability. The method shows great promise for use in protein structure prediction, determination, simulation, and design.

## Introduction

The prediction of a protein's structure from its amino acid sequence remains one of the greatest unsolved problems in computational molecular biology. The problem attracts much interest because it both is intellectually challenging and has important practical applications such as drug development and genome annotation.

According to Anfinsen's famous hypothesis, a protein's native structure is determined by its sequence and corresponds to minimal Gibbs energy [1]. Levinthal's paradox implies that a brute force enumeration of all possible conformations for a given sequence is both computationally and physically impossible [2]. This paradox is solved, at least in part, by the fact that the sequence introduces local structural bias, which narrows the conformational search space [3–6]. The native fold is thought to be the result of favorable local and long-range interactions [7,8]. As a consequence, protein structure prediction methods need two key ingredients: an energy function and an efficient method to explore the relevant parts of the conformational space associated with the sequence. The latter problem is considered to be the primary bottleneck in protein structure prediction today [9].

In practice, one first chooses a particular representation of a protein, ranging from a full-atom model to a C*α*-atom–only model. Based on the amino acid sequence of the protein, plausible protein-like conformations called decoys are generated. These decoys are subsequently accepted or rejected based on an energy function.

The strategy to generate decoys that are subsequently rejected or accepted comes in different flavors. One can generate a large set of decoys, and then select the decoy with the lowest energy [10–13]. The ROSETTA method generates decoys as part of a simulated annealing procedure to identify structures with minimum energy [9,14]. Markov Chain Monte Carlo (MCMC)–based methods [3,15,16] propose decoys that are accepted or rejected depending on their Boltzmann weights. The subject of this paper is the generation of decoys, that is, the exploration of the conformational space that is compatible with a given sequence. In particular, our goal is to generate decoys based on local sequence/local structure preferences [7,8], which we will refer to as “local structural bias.”

Recently, important progress in structure prediction was made due to the use of fragment libraries for decoy generation [9,14]. Fragment libraries consist of fragments derived from experimentally determined high-quality protein structures [17–20]. By combining fragments that are chosen based on sequence information, one can generate decoys that have a protein-like local structure [6,9,14]. The main idea behind the use of fragment libraries is to decrease the size of the vast conformational search space by taking local structural bias into account and using a finite set of fragments.

Despite the clear success of the fragment library approach, the method has some important shortcomings. The limited size of the Protein Data Bank (PDB) makes it very difficult to map a sequence stretch of even moderate length to a relevant set of structure fragments. Using fragment libraries in MCMC simulations is problematic because of their incomplete covering of the conformational space and nonprobabilistic nature [16,21]. Finally, the inherent discrete nature of fragment libraries conflicts with the continuous character of a protein's conformational space.

An important step forward was the HMMSTR method [22,23], which uses a Hidden Markov Model (HMM), trained from a fragment library [24], to predict local structure based on sequence. HMMSTR, and HMM-based approaches that do not include sequence information but are purely geometric [25,26], can be considered as probabilistic versions of fragment libraries.

Despite these advancements, probabilistic sampling in continuous space of plausible protein-like conformations that display realistic dihedral angles and secondary structure content is still an important unsolved problem [27–32]. A solution to this problem could have a profound effect on the success of protein structure prediction, design, and simulation [9].

Here, we provide such a solution by developing a probabilistic model that uses directional statistics to describe protein geometry in a natural, continuous space. The model makes it possible to sample plausible protein backbone conformations for a given sequence. We show that we readily generate near-native decoys for several proteins simply by enforcing compactness and self-avoidance, without using any additional energy terms. Our results thus support the view that relatively few compact structures are compatible with the sequence-encoded local structural bias [6], and provide the means to capture this bias in protein structure prediction, simulation, and design.

## Results/Discussion

### FB5–HMM: A Probabilistic Model of Local Protein Structure

**Figure 1:**
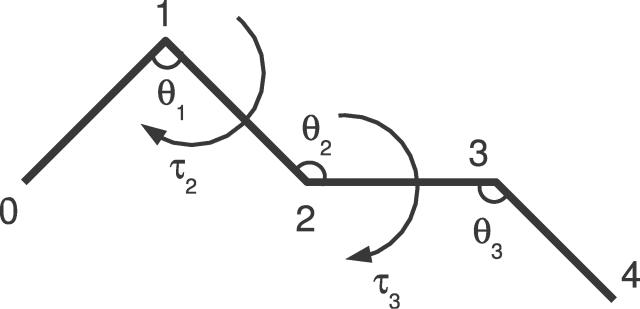
Schematic Representation of a Protein's C*α* Backbone The C*α* positions are numbered, and the pseudo bond angles *θ* and pseudo dihedral angles *τ* are indicated. The segment has length 5, and is thus fully described by two pseudo dihedral and three pseudo bond angles. The numbering scheme of the angles is chosen so that the angle pair (*θ_i_*,*τ_i_*), associated with position *i,* specifies the position of the C*α* atom at position *i* + 1.

Our goal is probabilistic sampling of plausible backbone conformations given a protein's sequence, and, optionally, given secondary structure information as well. A protein's backbone conformation, here taken to be characterized by the sequence of C*α* positions, can be effectively represented as a sequence of (*θ*,*τ*) angle pairs (Figure 1) [33,34]. Such a (*θ*,*τ*) sequence is equivalent to a sequence of unit vectors, each vector pinpointing the C*α* position of one amino acid (see Materials and Methods). Hence, a probabilistic model needs to be developed that allows sampling a sequence of unit vectors based on one or two sequences, respectively specifying amino acid type and secondary structure class (that is, helix, *β*-strand, and coil).

An HMM can deal with the sequential aspect of the problem [35], provided a way can be found to represent the unit vectors. A solution to this problem comes from the field of directional statistics, a branch of statistics that deals with probability distributions over orientations, directions, or angles [36]. Directional statistics has for example been applied to the modelling of wind directions and astronomical observations on the celestial sphere. To represent the unit vectors, we used the 5-parameter Fisher-Bingham (FB5) distribution [37], which is the equivalent on the sphere of the Gaussian distribution in the plane.

**Figure 2:**
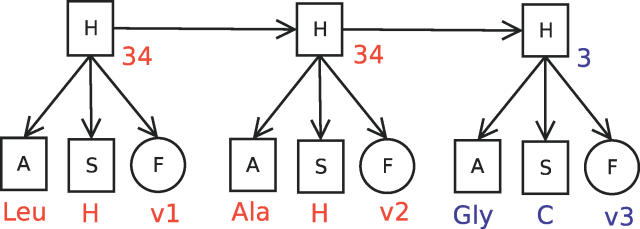
Conditional Dependency Graph of FB5–HMM Squares represent discrete nodes, circles represent the FB5 node with unit vector output. The arrows indicate conditional dependencies. Three slices are shown, corresponding to three consecutive amino acid positions. A possible set of node values is shown in color (v1, v2, and v3 are unit vectors). The hidden node sequence (34,34,3) corresponds to two C-terminal positions of an *α*-helix, followed by a coil residue. A, amino acid node; F, FB5 node; H, hidden node; S, secondary structure node.

Figure 2 shows the conditional dependency graph of an HMM (called FB5–HMM) that combines amino acid sequence, secondary structure, and detailed geometric information. Two discrete nodes, A and *S,* represent the 20 amino acid types and the three secondary structure classes, while the continuous node *F* represents the unit vector describing C*α* geometry. The three nodes *A, S,* and *F* are conditionally dependent on a hidden, discrete node *H*. That is, the hidden node value at a given sequence position specifies the probabilities of observing a specific amino acid type, secondary structure class, and unit vector at that position. The dependencies between the sequence positions are encoded in the transition probabilities of going from one hidden node value to another. Hence, FB5–HMM aims to capture the joint probability distribution of an amino acid sequence **A**, a secondary structure sequence **S**, and a sequence of unit vectors or angle pairs **X** describing the backbone geometry. The joint probability distribution is given by

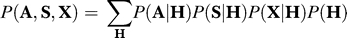
where the sum runs over all possible hidden node sequences **H**. In the trained model, each hidden node value ties together matching preferences for amino acid type, secondary structure, and local geometry. The use of an HMM with multiple outputs makes challenging operations such as sampling a set of backbone angles given an amino acid sequence computationally feasible.

**Figure 3:**
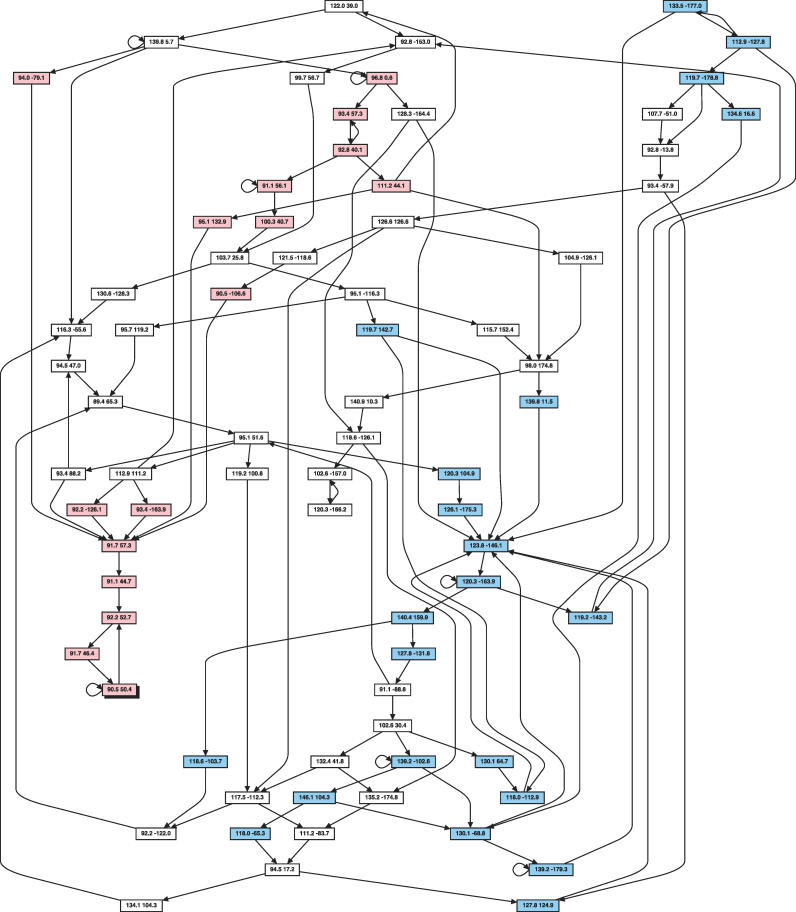
Transitions Occurring between (*θ*,*τ*) Angle Pairs in Proteins according to FB5–HMM The graph shows some of the most important possible hidden node transitions in FB5–HMM. Each hidden node value is represented as a box, showing the associated mean direction as a pair of (*θ*,*τ*) angles. For clarity, only a subset of all transitions is shown: for each hidden node value, the incoming and the outgoing transition with the highest probability is shown as an arrow. If one of them is a self-transition, the second best incoming or outgoing transition is also shown. Hidden node values mainly associated with *α*-helices are shown in light red, with *β*-strands in light blue, and with coils in white.

The optimal number of hidden node values (which is 75) and all other associated parameters of FB5–HMM were determined by training the HMM using a large set of representative protein structures (see Materials and Methods). Figure 3, which shows the most important transitions between the hidden node vales, gives an impression of the overall structure of FB5–HMM.

Nearly all hidden node values (73 out of 75) are associated with a strong preference (*P* > 0.8) for a single secondary structure class (helix = 16, *β*-strand = 21, coil = 36). The only amino acid types that are associated with a probability greater than 0.3 (given a hidden node value) are Gly and Pro, which reflects their special geometric preferences. The trained HMM is quite sparse: only 1,352 (24%) of all possible hidden node transitions occur with a probability above 0.0001. The parameters of FB5–HMM are available online as supporting information (Dataset S1).

The HMMSTR method [22,23] also uses an HMM approach to represent local structural bias, but makes use of a discretized representation of the full-atom protein backbone. Here, the final number of hidden node values was considerably higher (281), but the number of nonzero transitions was lower (371) than for FB5–HMM. It should be noted that HMMSTR was extensively manually optimized for prediction, while training of our model was fully automated.

### Generating Protein-Like Backbones

In this section, we show that FB5–HMM generates C*α* backbones with realistic, protein-like geometries, and briefly explain the sampling method.

To sample a C*α* backbone given an amino acid sequence, and optionally given a secondary structure sequence as well, a sequence of hidden node values needs to be sampled from FB5–HMM first. Once the hidden node values are sampled, it is trivial to sample a sequence of unit vectors describing a C*α* backbone (see Materials and Methods, and the example discussed below). The classic inference methods for HMMs, Viterbi path decoding, and posterior decoding [35], do not apply here because they are not aimed at *sampling* but *predicting*. However, the problem can be solved using Forward-Backtrack (FwBt) sampling, a little-used inference method previously used in gene finding [38]. Using FwBt sampling, it also becomes possible to resample the angles of a stretch of residues seamlessly. Note that the Forward-*Backtrack* algorithm (a sampling method) should not be confused with the related Forward-*Backward* algorithm (a method to calculate the posterior distribution) [35].

**Figure 4:**
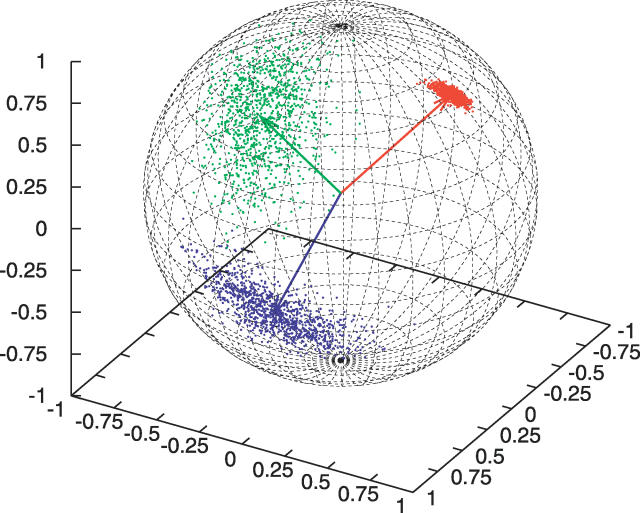
Three Point Sets Sampled from the FB5 Distribution on the Sphere The three sets consist of 1,000 unit vectors sampled from the FB5 distributions associated with hidden node values 3 (blue), 34 (red), and 44 (green), respectively These three node values are typical representatives of coil, *α*-helix, and *β*-strand geometry. The samples were plotted on the unit sphere, and the mean directions of the three FB5 distributions are indicated with arrows.

How a hidden node value specifies a probability distribution over unit vectors deserves some more explanation. Each hidden node value is associated with a set of parameter values for the FB5 distribution that specify its mean direction, shape, extent, and orientation. For example, in Figure 4, three sets of 1,000 points sampled from the FB5 distributions associated with hidden node values 3, 34, and 44 are shown on the unit sphere. These hidden node values are associated with coils, *α*-helices, and β-strands, respectively.

**Figure 5:**
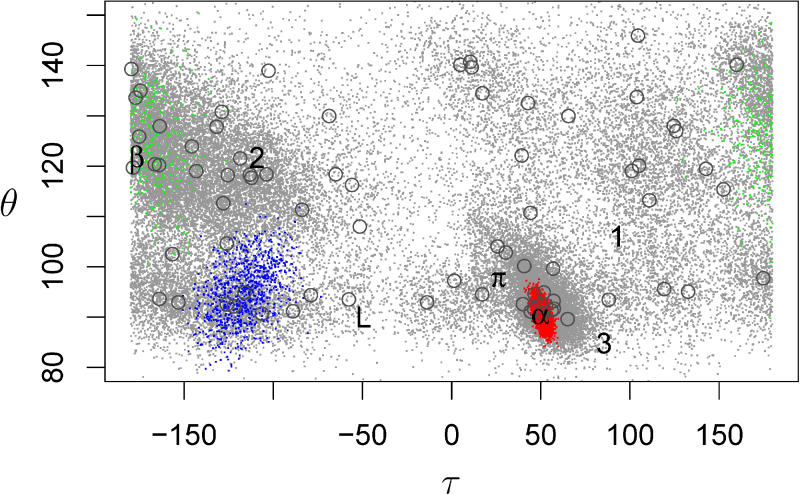
Scatter Plot of the (*θ*,*τ*) Angles in a Sampled Dataset The dataset consisted of 500 sequences of length 100 generated using FB5–HMM. The ideal (*θ*,*τ*) values of some conformations are indicated: *α*: *α*-helix, *β*: *β*-strand, π: π-helix, L: left-handed *α*-helix, 3: 3_10_-helix, 1 & 2: Poly-Proline helices types I and II. The open circles indicate the mean directions of the 75 FB5 distributions. Angle pairs generated by hidden node values 3, 34, and 44 are plotted in blue, red, and green, respectively. These three hidden node values are typical representatives of hidden node values that correspond to coil, *α*-helix, and *β*-strand geometry, respectively.

The entire (*θ*,*τ*) space accessible to proteins is covered by a mixture of 75 FB5 distributions, of which the 75 mean directions are shown in Figure 5. It should be noted that mean directions that are close together in the plot might belong to hidden node values that specify very different secondary structure class and amino acid type preferences.

A simple example will serve to explain the process of sampling a C*α* trace given a sequence. Suppose we want to sample a set of (*θ*,*τ*) angles given the sequence (Ala, Leu, Gly). In the first step, a hidden node sequence of length three is sampled using the FwBt method with the (Ala, Leu, Gly) sequence as input. Note that if a secondary structure assignment is given as well, the hidden node sequence can be sampled using both the amino acid sequence and the secondary structure sequence. A plausible hidden node sequence resulting from the sampling from the amino acid sequence (Ala, Leu, Gly) is, for example, (34,34,3). Examining these hidden node values shows that hidden node value 34 is associated with a high probability of emitting Leu and Ala as amino acid symbol and helix as secondary structure symbol, while hidden node value 3 is mainly associated with Gly and coil. Hence, the sampled sequence of hidden nodes corresponds to the two hydrophobic C-terminal residues of a helix, followed by a coil beginning with a Gly residue. Next, the (*θ*,*τ*) angle pairs are sampled from the FB5 distributions associated with hidden node values 34 and 3. The FB5 distributions associated with hidden node values 34 and 3 have (*θ* = 90.5,*τ* = 50.4) and (*θ =* 95.1,*τ =* 116.3) as mean directions, respectively. A possible sampled sequence of (*θ*,*τ*) angle pairs could be for example:

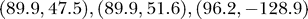
Note that the two first (*θ*,*τ*) pairs have values that are typical for an *α*-helix (Figure 5).

**Figure 6:**
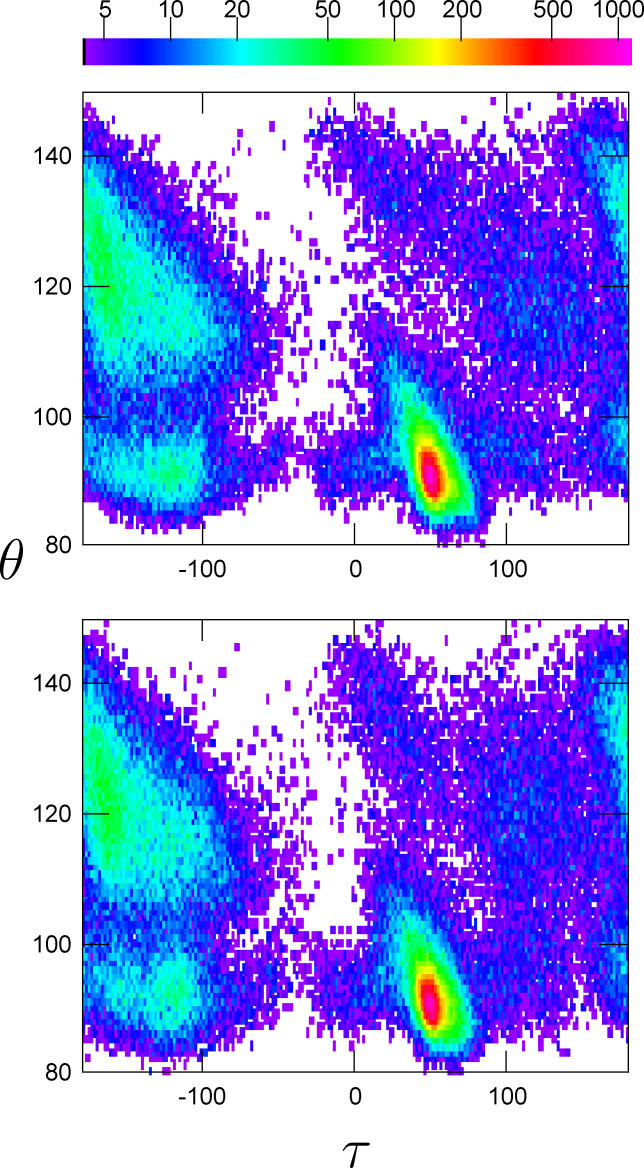
Histograms of the (*θ*,*τ*) Angle Pairs Histograms are shown for the training set (upper) and the decoy set (lower). The bin size is 1° × 1°. The color scale refers to the number of counts per bin. Bins with a count below 4 are white.

The FB5–HMM model correctly captures the distribution of pseudo bond and dihedral angles found in proteins. To show this, we sampled a set of backbone angle sequences with the same total number of residues as the dataset and constructed histograms of the (*θ*,*τ*) angles. The (*θ*,*τ*) plot can be considered as the C*α* equivalent of the classic Ramachandran plot [34,39]. The similarity of the resulting 2-D histograms, both in terms of overall shape and detailed angle pair frequencies, indicates that FB5–HMM accurately reproduces the (*θ*,*τ*) distribution found in real proteins (Figure 6).

### Secondary Structure Content

FB5–HMM not only captures the distribution of the (*θ*,*τ*) angles, but also their sequential dependencies, and as a consequence generates secondary structures that follow realistic length distributions. To show this, we generated a large set of decoys, and analyzed the lengths of their secondary structures.

For each protein in the dataset, a matching decoy with the same length was generated. Secondary structure was assigned using the program P-SEA [40]. This program only makes use of the C*α* coordinates and evaluates local geometry, which allowed us to use exactly the same secondary structure definitions for both dataset and decoys.

**Figure 7:**
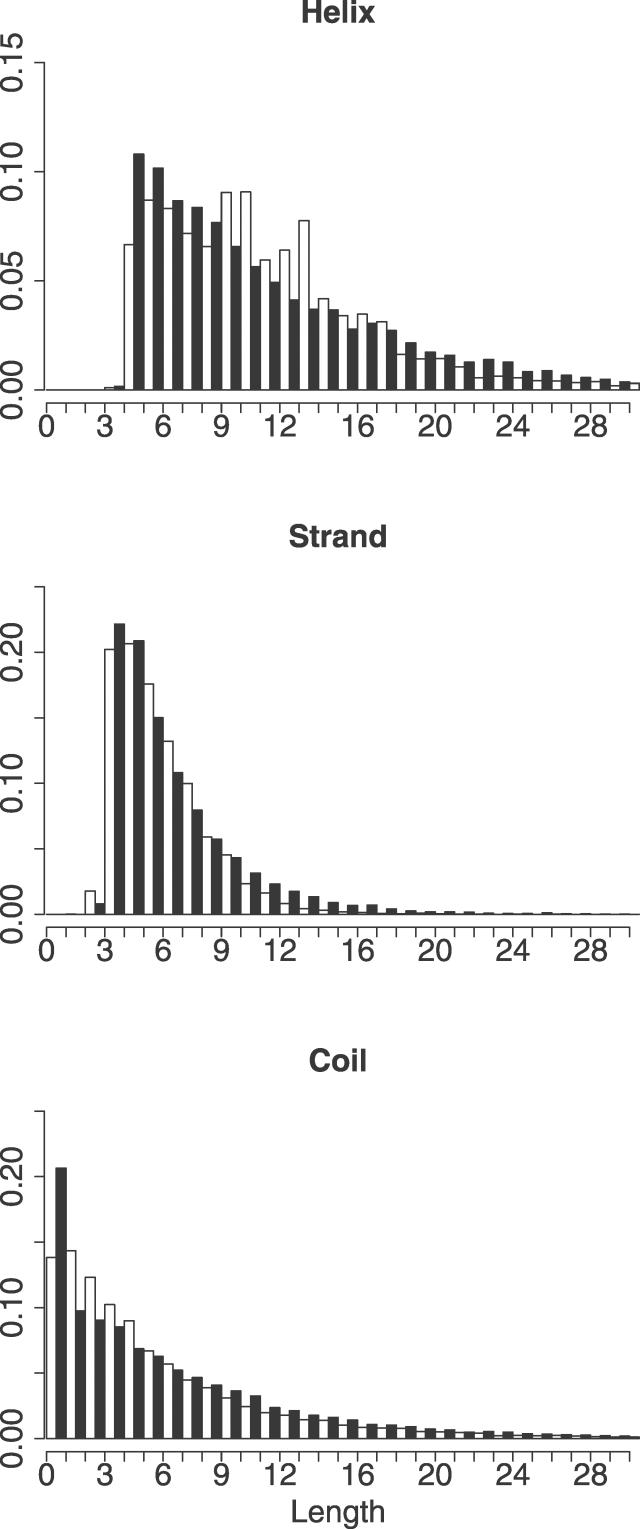
Histograms of Secondary Structure Element Length Histograms of the lengths of the secondary structure elements in the training set (white bars) and the decoy set (black bars).

The overall secondary structure content of the protein dataset and the decoy set are remarkably close to each other (helix, including *α*-helix, 3_10_-helix, and *π*-helix: 34% and 32%; *β*-strand: 25% and 24%; coil: 41% and 44%). Figure 7 shows the length distributions of helices, *β*-strands, and coils in the protein dataset and the decoy set. The length distributions of the secondary structures in the decoys closely match those in the protein structures, especially in the case of *β*-strands. As the generated decoys were not enforced to be compact, the secondary structure content cannot be ascribed to compactification effects that can give rise to extensive secondary structure formation in lattice [41] and tube [42,43] models.

The quality of the generated decoys was confirmed by constructing all-atom backbones from the C*α* coordinates using the program MAXSPROUT [44]. According to the DSSP program [45], which requires proper hydrogen bonding for secondary structure assignment, the dataset contains 32% helix (of which 30% is *α*-helix), which is identical to the percentage calculated by PSEA. Note that the same procedure does not apply to *β*-strands because DSSP requires interstrand hydrogen bonds to recognize *β*-strands and *β*-sheets, while our model is not meant to incorporate nonlocal interactions and hence does not bring *β*-strands together into *β*-sheets. However, manual inspection of the generated *β*-strands confirms they have the expected geometry, including the distinct right-handed twist observed in real proteins [46].

### Compact Decoys Using Sequence Information

We used FB5–HMM to generate compact decoys for six target proteins that were the subject of two previous studies [13,14]. Four of them are all-helical, while two consist of *α*-helices and a single *β*-sheet (Table 1).

**Table 1:**
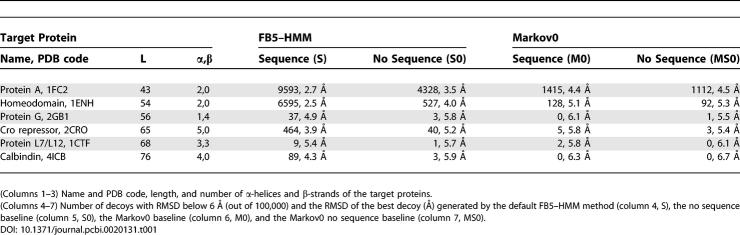
Generation of Compact Decoys Using Sequence Information

For each of these proteins, we generated 100,000 compact decoys using the radius of gyration (*R_g_*) of the target proteins (see Materials and Methods). Apart from compactness and self-avoidance, no additional energy terms were used. We consider decoys with a C*α*-based root mean square deviation (RMSD) with the native structure that is below 6 Å as “good” decoys [28]. We emphasize that the dataset used to train FB5–HMM did not contain any proteins that are homologous to the target proteins, or any proteins with a fold identical to that of a target protein.

To evaluate the results of the default FB5–HMM method that generates compact decoys using amino acid sequence information (Table 1, Method S), we used three baselines. The S0 baseline does not make use of amino acid sequence information for sampling the backbone angles. The Markov0 (M0) baseline uses the target sequence but uses a uniform transition matrix for the hidden nodes, which corresponds to removing the arrows between the hidden nodes in Figure 2. Finally, the MS0 baseline uses a uniform transition matrix and does not make use of sequence information.

The use of the S0 and M0 baselines is challenging, because they are expected to generate decoys that are much better than truly random decoys. The S0 baseline generates random compact decoys with a protein-like geometry, and is thus expected to generate protein-like folds by chance [47]. The M0 baseline generates decoys with realistic (*θ*,*τ*) angles for the given amino acid sequence, but neglects the dependencies between consecutive (*θ*,*τ*) pairs. The MS0 baseline simply generates random compact structures with (*θ*,*τ*) pairs that fall in the range that is allowed for proteins. The results are shown in Table 1.

**Figure 8:**
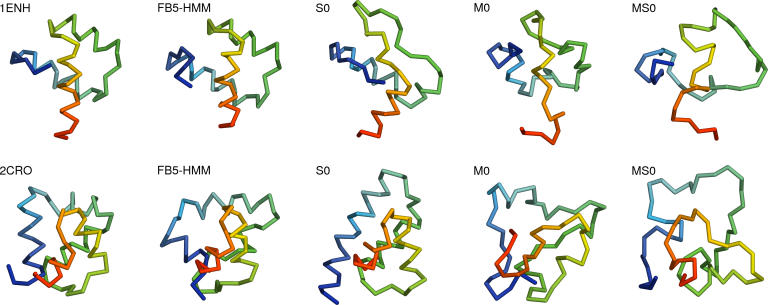
Best Compact Decoys Generated Using FB5–HMM The best compact decoys generated using sequence information (Table 1, S) are shown for 1ENH (top) and 2CRO (bottom). From left to right: crystal structure, FB5–HMM, S0 baseline, M0 baseline, MS0 baseline. The N-terminus is shown in blue. The figure was made with PyMol (DeLano Scientific, http://www.delanoscientific.com).

Strikingly, FB5–HMM generates good decoys for all targets (Table 1, S). The best decoys for targets 1ENH and 2CRO are shown in Figure 8. The number of good decoys ranges from almost 10% for the smallest helical target (1FC2) to several good decoys (37 and 9) for the targets containing a *β*-sheet (2GB1 and 1CTF). Given the fact that no energy function was used to handle the nonlocal interactions during decoy generation, besides enforcing compactness and absence of steric clashes, this result is quite remarkable.

Indeed, the (modest) success for the *β*-sheet containing targets is noteworthy since our model does not incorporate the long-range interactions that assemble *β*-strands into *β*-sheets. In general, proteins containing *β*-sheets are challenging targets [13,14].

The S0 baseline, which does not take the target sequence into account, generates significantly fewer good decoys for all targets (Table 1, S0). In addition, the RMSD between the best decoy and the native structure is consistently higher for the S0 baseline than for FB5–HMM. Both FB5–HMM and the S0 baseline generates compact, protein-like decoys. Since FB5–HMM performs significantly better than the S0 baseline, we can conclude that the model successfully captures at least a significant part of the local structural bias encoded in an amino acid sequence.

The M0 baseline performs much worse than FB5–HMM for all targets and does not generate a single good decoy for two of them (Table 1, M0, targets 2GB1 and 4ICB). This points out that the sequential dependencies between the (*θ*,*τ*) angle pairs are extremely important for obtaining good decoys. Surprisingly, it is even better to neglect target sequence information (S0 baseline) than to neglect the dependencies between the angles (M0 baseline).

In fact, the M0 baseline does not seem to perform significantly better than the MS0 baseline, which essentially only depends on the length of the protein (Table 1, MS0). Both the M0 and MS0 baseline produce decoys that do not resemble proteins because they lack realistic secondary structure content (Table 2). Indeed, all “good” M0 and MS0 decoys have a very low helix and strand content (more than 90% of the residues are coil), which corresponds to the low helix and strand content reported for random compact polymer conformations [48–50], when the polymers are not represented as tubes with a certain thickness [42,43] or confined to a lattice [41]. This is in strong contrast to the decoys generated by FB5–HMM and the S0 baseline (Figure 8, Table 2), which again emphasizes the importance of taking the sequential angle dependencies into account.

**Table 2:**
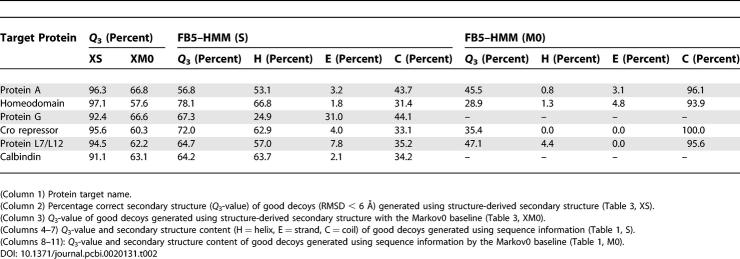
Secondary Structure Content of the Good Decoys

**Table 3:**
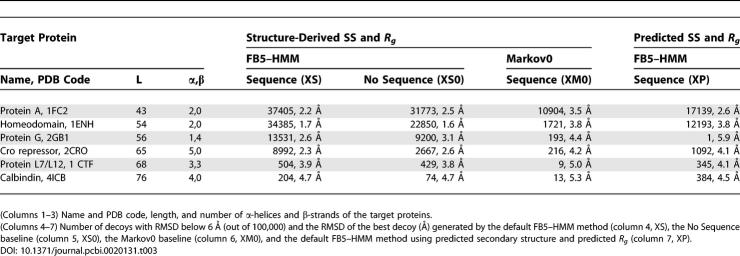
Generation of Compact Decoys Using Sequence and Secondary Structure Information

### Adding Secondary Structure Information

One of the great advantages of incorporating secondary structure information into the model is that sampling from the conformational space associated with both an amino acid sequence and a secondary structure sequence becomes possible. The latter sequence could come from a prediction algorithm, but also from experimental data.

To show that our model incorporates secondary structure information successfully, we sampled compact decoys using both amino acid sequence and secondary structure information derived from the native structure (Table 3, XS). Again, we make use of two baselines. The XS0 baseline makes use of secondary structure, but not sequence. The XM0 baseline includes secondary structure and sequence, but uses a uniform transition matrix and hence neglects the dependencies between consecutive (*θ*,*τ*) pairs.

As expected, and as was previously shown for a fragment assembly method [19], structure information indeed boosts the generation of close-to-native decoys dramatically (Table 3, XS). The most impressive improvement occurs for 2GB1, where including secondary structure information increases the number of good decoys from 0.037% to 13.5%. In addition, the RMSD of the best decoy improves considerably in all but one case. For all targets, more than 90% of the residues in the good decoys have the correct secondary structure (Table 2).

Two additional observations deserve to be highlighted. Even in the presence of secondary structure information, sequence information matters (Table 3, compare XS and XS0). Clearly, FB5–HMM does more than simply translating secondary structure into bond angles. Rather, secondary structure information narrows the sampling space but leaves ample room for inference of local structural bias.

A second observation is that even in the presence of secondary structure information, the Markov0 model (baseline XM0) still performs much worse than FB5–HMM (XS) and the XS0 baseline. This implies that the sequential dependencies of the (*θ*,*τ*) angles remain important even within the constraints of a secondary structure assignment. In fact, generating compact decoys using sequence only (Table 1, S) generally performs better than using both secondary structure and sequence information but neglecting the sequential dependencies of the angles (Table 3, baseline XM0). This is true in terms of the number of good decoys generated, the RMSD values of the best decoys, and the secondary structure similarity to the native structure (Table 2).

The question naturally arises whether noisy secondary structure and *R_g_* information, for example derived from predictions, still improves decoy generation. To address this question, we generated decoys using predicted secondary structure and predicted *R_g_*.

**Figure 9:**
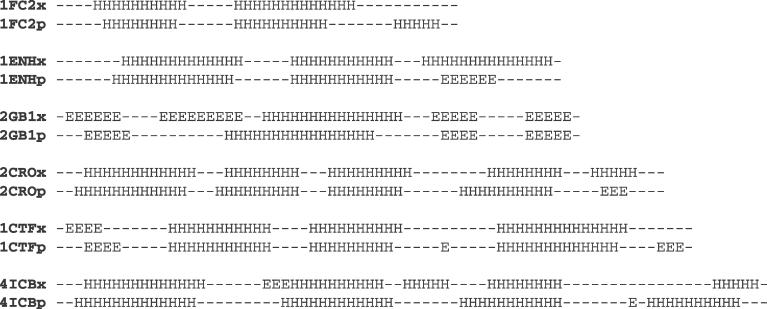
Secondary Structure of the Target Proteins (First line) Secondary structure assignment derived from the crystal structure. (Second line) Predicted secondary structure assignment.

Using sequence, predicted secondary structure information, and predicted *R_g_* (Table 3, XP) generates more good decoys than using sequence and structure-derived *R_g_* only (Table 1, S). In most cases, this comes at the expense of a higher RMSD for the best decoys, presumably due to the secondary structure prediction errors. The bad performance in the case of 2GB1 is probably due to the prediction of one of the *β*-strands as coil (Figure 9). Hence, FB5–HMM provides a convenient way to shuttle secondary structure prediction results into 3-D structure prediction methods.

### Comparison with Two Fragment Assembly Methods

We have used the same target proteins as two previous studies that focus on decoy generation using fragment assembly methods [13,14]. Both studies also use the same criteria for good decoys, that is, having a C*α*-based RMSD below 6 Å with the native structure. As a result, we can directly compare our results with the results reported in these two studies.

The fragment assembly method in the first study forms the basis of the ROSETTA de novo protein structure prediction method [14,51]. Fragments are selected based on multiple sequence information, and assembled into decoys using a simulated annealing procedure and a probabilistic nonlocal energy function. A direct comparison of the two methods is of course extremely unfair, since FB5–HMM is a local structure sampling method, while ROSETTA is a complete structure prediction method incorporating nonlocal interactions. In addition, ROSETTA has a clear advantage because it uses multiple sequence information in the selection of the fragments, while FB5–HMM only uses a single sequence. Nonetheless, the comparison offers some interesting insights.

As expected, the percentage of good decoys is much higher for ROSETTA than for FB5–HMM for most target proteins (Table 4). However, in some respects FB5–HMM clearly performs better. First, ROSETTA does not generate a single good decoy in the case of Protein G, while FB5–HMM does produce good decoys. Second, the RMSD values of the best decoys are in general lower for FB5–HMM than for ROSETTA. Hence, in this view, generating a large set (100,000) of compact decoys using FB5–HMM leads to better results than carefully predicting relatively few (100) candidate structures using ROSETTA, at least according to the results given by Simons et al. [14] for these six small target proteins. This is an important point, as combining a fairly inaccurate, but computationally cheap method to generate decoys with an accurate, but computationally expensive method to identify and refine promising structures has recently led to considerable success [9].

**Table 4:**
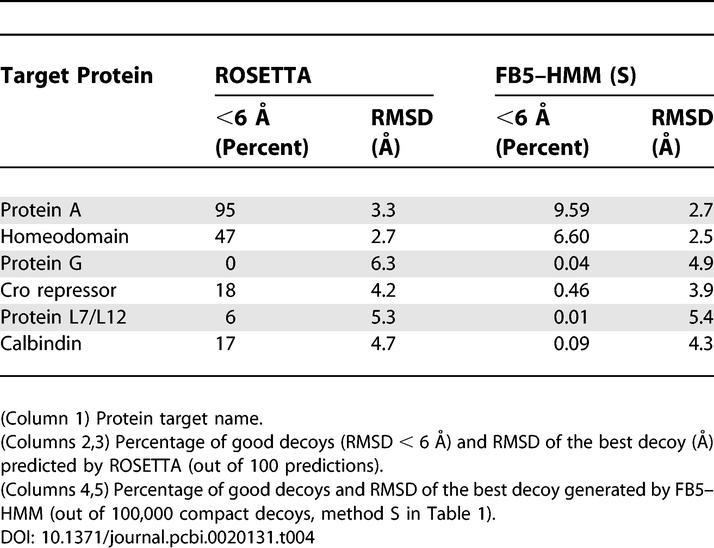
Comparison of FB5–HMM and ROSETTA

The second study we use for comparison describes a fragment assembly method that uses secondary structure information derived from the true structure to produce compact decoys [13]. This method does not make use of sequence information, but only secondary structure information. The results of this study can thus be directly compared with those produced by FB5–HMM using secondary structure information, but not sequence (Table 3, XS0). Table 5 compares both methods for the four common target proteins. FB5–HMM performs considerably better than the fragment assembly method, judging by the number of good decoys and the RMSD values of the best decoys.

**Table 5:**
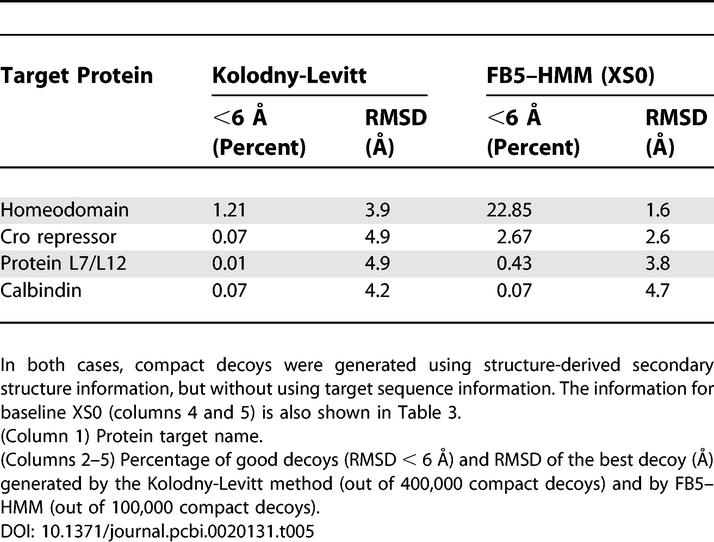
Comparison of FB5–HMM and the Kolodny-Levitt Fragment Assembly Method

### Conclusions

We described a probabilistic model that can be used to sample C*α* backbones based on a protein's amino acid sequence, incorporating secondary structure information if available. The method is conceptually elegant, has excellent computational complexity [38], and handles in principle any sequence or fragment length. The generated decoys have protein-like geometry, reflected in realistic angle and secondary structure length distributions.

The potential applications of FB5–HMM are numerous. In de novo protein structure prediction, the use of local structural bias can avoid generating misfolded conformations that are due to an imperfect energy function [6]. In homology modelling, FB5–HMM could guide the construction of variable loops [52,53]. Because of the probabilistic nature of the model, it can be used to propose local conformational changes that respect the detailed balance condition [16,21], making it possible to estimate thermodynamic averages using MCMC simulations [54]. In experimental methods such as NMR, X-ray crystallography, or Small Angle X-ray Scattering, the model could be used to generate conformations that take the local structural bias and the experimental data into account [55–57]. By inference of optimal sequences for a given backbone conformation, for example using Viterbi decoding [35], FB5–HMM could also be helpful in fold recognition [58] or protein design [59,60].

The model could in principle be extended in several ways, including using Dirichlet nodes to incorporate multiple sequence information [61], explicitly modelling the length distributions of the secondary structure elements [62] or adding additional hidden nodes and dependencies. A model with a very similar architecture would make an excellent probabilistic model of the full backbone structure of proteins, provided a tractable distribution to represent the joint probability distribution of two dihedral angles (that is, a distribution on the torus) is available. Preliminary results obtained using a bivariate von Mises distribution [63] confirm this approach is indeed quite feasible. Many of the extensions mentioned above pose considerable computational and statistical challenges, and will be the subject of future studies.

Surprisingly, FB5–HMM readily generated native-like decoys for several proteins when merely self-avoidance and compactness were enforced. Our results thus support the view that the native fold of a protein is at least partly encoded by the local structural bias associated with its amino acid sequence [3–6,15]. Recently, it was suggested that only relatively few compact structures are compatible with the local structural bias imposed by a protein's amino acid sequence [6]. Our results are in accordance with this, and also point out the importance of the detailed sequential dependencies of the backbone angles, even within the constraints of a given secondary structure assignment.

## Materials and Methods

### C*α* backbone parameterization.

The C*α* backbone of a protein can be considered as a string of beads (Figure 1), in which each bead corresponds to the C*α* atom of an amino acid. Since the distance between two consecutive C*α* atoms in a protein can be considered constant (3.8 Å), the conformation of the C*α* backbone can be described using a sequence of pseudo angles and pseudo dihedral angles [33,34], called *θ* and *τ*, respectively (Figure 1). The term *pseudo* points to the fact that the consecutive C*α* atoms are not actually connected by a single chemical bond. In proteins, the angle *θ* lies in [80,150], while the dihedral angle *τ* can adopt all values in [−180,180].

The conformation of *n* C*α* atoms is fully described by *n* − 2 pseudo angles and *n* − 3 pseudo dihedral angles. Adding one C*α* atom to a given C*α* backbone corresponds to adding one (*θ*,*τ*) pair. Hence, the geometry of *n* C*α* atoms can be described by *n* − 2 (*θ*,*τ*) angle pairs, where each angle pair positions one C*α* atom. Note that the first three C*α* positions are fixed by the first *θ* angle, and that the first *τ* angle can be ignored.

Each (*θ*,*τ*) pair is conveniently represented as a unit vector **v** = (*x*,*y*,*z*) (that is, a point on the unit sphere), simply by interpreting the (*θ*,*τ*) pair as a set of polar coordinates:

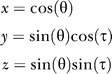
Hence, the C*α* backbone of a protein can also be encoded as a sequence of unit vectors.

### The Fisher-Bingham distribution.

We use the FB5 distribution [37] to create probability distributions over unit vectors. FB5 is the analogue on the unit sphere of the bivariate normal distribution with an unconstrained covariance matrix.

The probability density function of the FB5 distribution is given by:

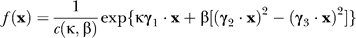
where **x** is a unit vector and c(*κ*,*β*) is a normalizing constant [37]. The parameter *κ* (with *κ* > 0) determines the concentration or spread of the distribution, while *β* (with 0 ≤ 2*β* < *κ*) determines the ellipticity of the contours of equal probability. The higher the *κ* and *β* parameters, the more concentrated and elliptical the distribution will be, respectively. Vector ***γ***_1_ is the mean direction, and vectors ***γ***_2_, ***γ***_3_ are the major and minor axes. The latter two vectors **γ** determine the orientation of the equal probability contours on the sphere, while the first vector determines the common center of the contours.

### Training FB5–HMM using protein data.

Parameter learning for FB5–HMM was done using Stochastic Expectation-Maximization (S-EM) [64,65]. Briefly, S-EM consists of the following steps. First, the FB5–HMM is initialised using random parameters and hidden node values. In the Expectation step, the values of the hidden nodes are filled in using a single sweep of Gibbs sampling, while the values of the observed nodes are kept fixed [65]. In the Maximization step, the filled-in values of the hidden nodes are used to update the FB5–HMM's parameters. Advantages of S-EM compared with classic deterministic learning methods include less dependence on the starting parameters of the model and a lower chance of getting stuck in local maxima [64].

Choosing an appropriate hidden node size is vital for the success of the model. If the size is too low, the model will be too coarse, while if the size is too high, it will lead to overfitting. We estimated the ideal hidden node size using the Integrated Completed Likelihood Criterion (ICL) [66], an entropy penalized version of the Bayesian Information Criterion [67,68] :

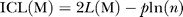
where *L*(*M*) is the logarithm of the likelihood (LogLik) of the completed data after convergence, *M* is the hidden node size, *p* is the number of parameters of the model, and *n* is the number of observations. The ICL value reaches a maximal value for the best model.

We trained FB5–HMM using 1,428 protein domains, all belonging to different superfamilies, from the SABmark dataset, version 1.65 [69]. The list of structures is available in Dataset S2. Secondary structure was assigned using P-SEA [40]. P-SEA assigns secondary structure (helix, *β*-strand, and coil) based on C*α* coordinates only, which allowed us to use the program on the full backbone structures in the training set and the C*α*-only decoys. The training set contained information for 228,842 residues.

**Figure 10:**
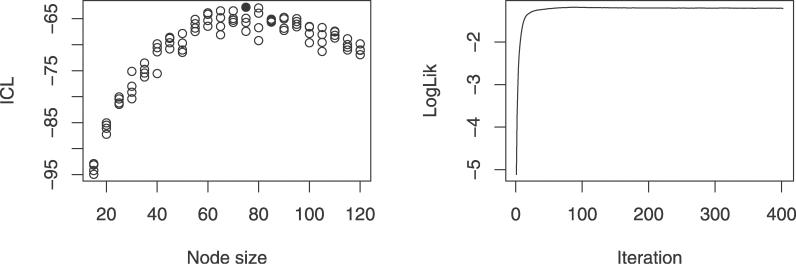
Training FB5–HMM (Left) ICL plotted versus hidden node size. For each hidden node size, four models were trained. The ICL reaches a maximum for one of the models with a hidden node size of 75 (indicated with a solid dot). (Right) Evolution of the LogLik of the completed data during training. The LogLik is plotted against the number of EM iterations.

The ICL was calculated for hidden node sizes 15 to 120 (with a step size of 5), using the LogLik obtained after convergence of the S-EM algorithm (Figure 10). For each node size, the training was repeated four times with different starting conditions to lower the chance of picking a model that got stuck in a local minimum. For a model with a hidden node size of 75, resulting in an HMM with 7,800 parameters, the ICL value reached its maximum value. It is this model that is used in the article. The parameters of the model are available in Dataset S1.

### Sampling a C*α* backbone.

FB5–HMM (Figure 2) has one discrete hidden node *H,* two discrete nodes *A*,*S*, and one continuous node *F*. The three nodes *A*,*S*,*F* represent the amino acid type, the secondary structure class, and the unit vector at a given sequence position, respectively.

FB5–HMM can be used to generate a sequence of unit vectors given an amino acid sequence **A** = *a*_0_,…,*a_L_*_−1_ and secondary structure sequence **S**, if available. Each unit vector corresponds to one (*θ*,*τ*) pair, and specifies the position of one C*α* atom. For simplicity, we will assume amino acid information only here.

The problem can be reduced to sampling a sequence **H** of hidden node values conditional on the amino acid sequence **A**. Once the hidden node sequence is sampled, a sequence of vectors describing the backbone can be obtained as follows. A hidden node value *h* at position *l* in **H** specifies a parameter set (*κ*,*β*,*****γ*****_1_,***γ***_2_,***γ***_3_)*^h^* for node *F* at that position. Hence, a unit vector **v** at position *l* can be generated by sampling from the FB5 distribution [37,70] using the parameters that are specified by *h*.

Hidden node sequences **H** can be sampled from *P*(**H**|**A**) using the FwBt algorithm [35,38]. Note that this algorithm should not be confused with the related Forward-Backward algorithm used in posterior decoding. The FwBt algorithm is, in contrast to the Viterbi and posterior decoding algorithms [35], not widely used. Therefore, we describe its application to FB5–HMM here in some detail.

Essentially, the method calculates the forward variables [35] and performs a stochastic backtrack. The forward variables *f_h_*(*l*), which represent the probability of hidden node value *h* at position *l* given the amino acid sequence segment *a*_0_,…,*a_l_*, are recursively calculated as follows:

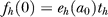


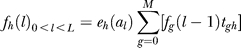
where *e_h_*(*a_l_*) is the emission probability of amino acid type *a_l_* given hidden node value *h*, *t_h_* is the probability of hidden node value *h* at position 0, *t_gh_* is the transition probability for hidden node values *g* and *h,* and *M* is the maximum hidden node value (which is equal to 75 for FB5–HMM).

To start the stochastic backtrack, a hidden node value *h* is sampled for the final sequence position *L* − 1, proportional to *f_h_* (*L* − 1). The backtrack is then continued recursively for the previous positions by sampling hidden node value *g* at position *l* proportional to *f_g_*(*l*)*t_gh_*, where *h* is the hidden node value at position *l* + 1. From the sampled hidden node sequence **H**, a sequence of unit vectors (and corresponding angle pairs) can then easily be sampled as described above.

### Resampling a segment of the backbone.

Given a previously sampled C*α* backbone (and a corresponding sequence of hidden node values), the FwBt algorithm can be used to resample a segment of the backbone. This corresponds to “rebuilding” a part of the structure seamlessly, which has important applications in MCMC simulations of proteins [16,21].

Starting from the previously sampled hidden node sequence **H**, a segment **H_s_** *^j:k^* from position *j* to *k* in **H** is selected and filled in with new hidden node values using the FwBt algorithm. In particular, the segment **H_s_** *^j:k^* is resampled conditioned on the amino acid sequence **A**, and the hidden node sequence segments from the start of the sequence to *j −* 1 and from *k* + 1 to the end of the sequence:

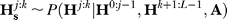
Let *p*,*q* be the hidden node values at positions *j* − 1, *k* + 1, respectively. First, we calculate the forward variables from *j* to *k*:

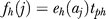


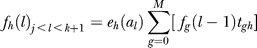
Backtracking starts at position *k* by sampling *h* proportional to *f_h_*(*k*)*t_hq_*, and continues recursively from *k* − 1 to *j*, by sampling *g* at position *l* proportional to *f_g_*(*l*)*t_gh_*, where *h* is the hidden node value at position *l* + 1. Once the hidden nodes in the segment are filled in, the (*θ*,*τ*) angle pairs in the segment from *j* to *k* are sampled as before, while the angle pairs outside the segment remain unaltered. How this application of the FwBt algorithm, which we call FwBt resampling, is used for compact decoy generation is explained in the next section.

### Generating compact decoys.

Generating compact decoys without steric clashes involves three steps: initialization, steric clash removal, and collapse. First, a sequence of angles is sampled using FwBt sampling and a corresponding C*α* backbone is constructed. In the next step, any steric clashes (defined as two C*α* atoms that are closer than 4.0 Å from each other) are iteratively removed by FwBt resampling of random stretches of the sequence and only accepting structures that diminish the number of steric clashes. Positions and lengths (from 1 to 15) of the segments to be resampled were chosen at random.

Once the steric clashes are removed, the structure is collapsed in a greedy way to produce a compact conformation. Random stretches are resampled as before, and the corresponding structure is accepted if the *R_g_* is lower than or equal to that of the previous structure. C*α* backbones that contain steric clashes are rejected. The collapse stage is stopped when the *R_g_* value falls below a given threshold (predicted or structure-derived *R_g_*) or after a maximum number of iterations (set to 1,000). Secondary structure was predicted using JPRED [71]. The predicted *R_g_* was calculated from the length *L* of the protein [72]:


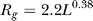


## Supporting Information

Dataset S1 Emission and Transition Parameters of FB5–HMM (291 KB TXT)

Dataset S2 Structures Used in Training FB5–HMM (11 KB TXT)

## Abbreviations

FB5: 5-parameter Fisher-Bingham distribution
FwBt: Forward-Backtrack
HMM: Hidden Markov Model
ICL: Integrated Completed Likelihood Criterion
MCMC: Markov Chain Monte Carlo
PDB: Protein Data Bank
R_g_: radius of gyration
RMSD: root mean square deviation
S-EM: Stochastic Expectation-Maximization
